# Harmonic Infrared and Raman Spectra in Molecular Environments Using the Polarizable Embedding Model

**DOI:** 10.1021/acs.jctc.0c01323

**Published:** 2021-05-19

**Authors:** Karen Oda Hjorth Minde Dundas, Maarten T. P. Beerepoot, Magnus Ringholm, Simen Reine, Radovan Bast, Nanna Holmgaard List, Jacob Kongsted, Kenneth Ruud, Jógvan Magnus Haugaard Olsen

**Affiliations:** † Hylleraas Centre for Quantum Molecular Sciences, Department of Chemistry, UiT The Arctic University of Norway, N-9037 Tromsø, Norway; ‡ Hylleraas Centre for Quantum Molecular Sciences, Department of Chemistry, University of Oslo, N-0315 Oslo, Norway; § Department of Information Technology, UiT The Arctic University of Norway, N-9037 Tromsø, Norway; ∥ Department of Chemistry and the PULSE Institute, Stanford University, 94305 Stanford, California, United States; ⊥ SLAC National Accelerator Laboratory, 94025 Menlo Park, California, United States; # Department of Physics, Chemistry and Pharmacy, University of Southern Denmark, DK-5230 Odense M, Denmark

## Abstract

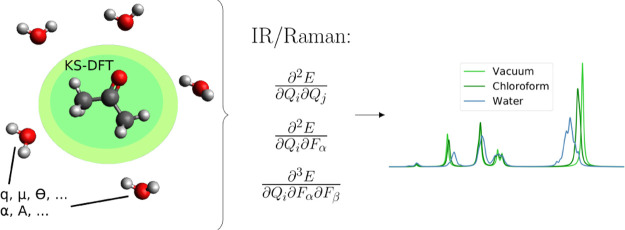

We present a fully analytic approach to calculate infrared (IR) and Raman spectra of molecules embedded in complex molecular environments modeled using the fragment-based polarizable embedding (PE) model. We provide the theory for the calculation of analytic second-order geometric derivatives of molecular energies and first-order geometric derivatives of electric dipole moments and dipole–dipole polarizabilities within the PE model. The derivatives are implemented using a general open-ended response theory framework, thus allowing for an extension to higher-order derivatives. The embedding-potential parameters used to describe the environment in the PE model are derived through first-principles calculations, thus allowing a wide variety of systems to be modeled, including solvents, proteins, and other large and complex molecular environments. Here, we present proof-of-principle calculations of IR and Raman spectra of acetone in different solvents. This work is an important step toward calculating accurate vibrational spectra of molecules embedded in realistic environments.

## 1 Introduction

Vibrational spectroscopy, in particular infrared (IR) absorption and Raman scattering, is one of the most important spectroscopic methods for elucidating molecular structure.^1^ Many vibrational bands primarily signify the presence of certain chemical bonds and functional groups. However, the so-called fingerprint region, located between 1500 and 500 cm^–1^, has in addition a spectral pattern that is often unique or near-unique for any given molecule, typically containing bands whose corresponding vibrational motions involve the backbone of the molecular structure. Although the use of databases of vibrational spectra of known compounds was essential in facilitating structural characterization of molecules in earlier days, this has in more recent years been complemented with a direct comparison to spectra obtained from quantum-chemical calculations.^2−4^

In the harmonic approximation, vibrational normal modes and their energy levels—determining the position of spectral peaks—are obtained from the second-order geometric derivatives of the molecular energy with respect to nuclear displacements. Spectral intensities, on the other hand, are found from the normal-mode displacement gradient of the relevant polarization properties, which for IR absorption is the electric dipole moment and for Raman scattering is the electric dipole–dipole polarizability.^5^ From a computational perspective, an added challenge in the calculation of vibrational properties compared to, for instance, properties involving only electric-dipole perturbations^6^ is the dependence of the basis functions on the nuclear positions.^2,4^ The theory and implementations of analytic first-^7^ and second-order^8^ geometric derivatives of molecular energies were presented already in the late 1960s and 1970s, respectively. These developments, and in particular the analytical calculation of second-order geometric derivatives at the level of density functional theory (DFT),^9−11^ have today made quantum-chemical calculations an integral part of structural characterizations of molecules using vibrational spectroscopy. At the electron-correlated levels of theory, coupled-cluster methods now allow vibrational frequencies to be obtained with an accuracy that rivals that of even highly accurate experimental studies.^12−14^ In addition, computationally elaborate schemes have been developed that allow anharmonicities to be efficiently calculated also at the DFT level of theory.^3,15,16^ More recently, IR and Raman spectra have also been calculated from Car–Parrinello molecular dynamics (MD) simulations, in which anharmonic effects as well as broadening of peaks due to solvent interactions are automatically included.^17−19^

Vibrational spectroscopy is also an important tool to understand molecular bonding and the interaction of molecules with their surroundings. Even small inter- or intramolecular interactions may change the strength of particular bonds and thus the corresponding vibrational frequencies. Hydrogen bonding may have a particularly significant effect on bond strengths as well as on the coupling to other nuclei through its strongly directional nature.^20,21^ Thus, vibrational spectroscopy is well-suited to study subtle interactions even in large biomolecular aggregates.^22^ This calls for computational methods that can model the effects of a surrounding environment.

Solvent effects are nowadays routinely included, for instance, through the use of continuum solvation models in calculations of vibrational spectra.^23^ In particular, the polarizable continuum model (PCM)^24,25^ is a popular approach used in quantum-chemical studies of solvated systems. However, specific intermolecular interactions are not described using such models. Moreover, modeling highly heterogeneous environments, such as proteins and other typical biomolecular systems, is problematic within a continuum approach. Quantum mechanics/molecular mechanics (QM/MM) methods, pioneered by Warshel and Levitt,^26^ are an appealing alternative. In QM/MM, the chemically interesting part of a system is treated at a quantum-mechanical level of theory and the surroundings are treated using a classical MM force field. Unlike in the PCM, such QM/MM methods retain the atomistic structure of the environment, which is important for describing directional and structural effects on molecular properties. A wide range of different QM/MM methodologies has been developed.^27−29^ They can be divided into three main classes depending on the level of approximation for the quantum–classical interactions, namely, mechanical, electrostatic, and polarized embedding. In mechanical embedding, the interactions between the quantum and classical parts are described purely classically. In this approximation, there are only corrections to the energy and indirect geometric effects from the environment. The quantities needed for simulating IR and Raman spectra can thus be computed using the same approaches as for pure QM and MM. The next level of complexity is electrostatic embedding, where the electronic density of the quantum part is directly polarized by the charge distribution of the classical part, that is, by the embedding potential. This is achieved through an embedding-potential operator that contains the electrostatic potential from the partial point charges (or more generally by the permanent multipoles) describing the charge distribution of the classical part. Finally, in polarized embedding, the classical part is described by a polarizable potential that thus allows for mutual polarization between the quantum and classical parts.

Second-order geometric derivatives within an electrostatic-embedding QM/MM approach were presented by Cui and Karplus.^30^ They used the full Hessian to perform a vibrational analysis of the entire system, that is, including both the QM and MM subsystems. The full vibrational analysis of such large systems may lead to computational bottlenecks in solving the vibrational eigenvalue problem due to the large matrices that would need to be diagonalized, as well as due to the high density of vibrational states. Various approaches have been proposed to deal with these challenges.^31−36^ Li and Jensen^37^ applied a partial Hessian vibrational analysis (PHVA)^38,39^ to the effective fragment potential^40,41^ method, which is a polarized-embedding approach, using numerical differentiation to determine the Hessian for the quantum part. The PHVA approximation in a QM/MM setting usually implies that only the QM–QM block of the full Hessian is used, thus ignoring the MM–MM, QM–MM, and MM–QM blocks. More recently, Lipparini et al.^42^ presented analytic second-order derivatives for a polarized-embedding approach based on fluctuating charges^43,44^ (QM/FQ) and used this to compute IR spectra, and later, this model was also used to calculate Raman spectra,^45^ in both cases within the PHVA approximation. Giovannini et al.^46^ derived and implemented second-order derivatives to their extended fluctuating charges and dipoles (QM/FQFμ) model.

In this work, we present the theory and implementation of fully analytic first- and second-order geometric derivatives of energies and first-order geometric derivatives of dipole moments and dipole–dipole polarizabilities in the framework of the polarizable embedding (PE) model and within the PHVA approximation.^47,48^ This work builds on our previous work on analytic first-order geometric derivatives of the PE energy.^49^ The PE model can be characterized as fragment-based classical embedding akin to QM/MM, with the difference that it focuses solely on the central quantum part. In this model, the environment is represented by fragment-based distributed multipoles and polarizabilities. It can be used to model complex systems, such as solute–solvent systems and large biomolecules (e.g., proteins and nucleic acids), as well as other large molecular systems that are amenable to fragmentation.^50^ To split large molecules into smaller fragments, the molecular fractionation with conjugate caps^51,52^ procedure can be used. The environment is treated classically, however, the parameters (multipoles and polarizabilities) can be derived from first-principles calculations on each individual fragment in the environment. This approach has been shown to yield highly accurate embedding potentials.^53−57^

The theory and implementation presented here build on earlier work, providing us with a flexible framework for the calculation of frequency-dependent molecular properties of arbitrary order for perturbation-dependent basis sets.^2,58−63^ The additional contributions arising from the PE model for the calculation of molecular Hessians as well as dipole and polarizability gradients have been implemented so as to be used together with the general open-ended framework of OpenRSP.^58,59,64^ The stage is thus set for extensions to higher-order geometric derivatives. Furthermore, the theory has been formulated in terms of the atomic-orbital (AO) density matrix, making the approach agnostic to the exact parametrization of the self-consistent field (SCF) wave function.^65^

Compared to the QM/FQ and QM/FQFμ models, our approach differs in two main directions. First, as described above, the environment is parametrized in terms of open-ended fragment-based permanent multipoles and multipole–multipole polarizabilities (the latter giving rise to induced multipoles), rather than fluctuating charges (and dipoles). Moreover, the multipoles and polarizabilities can be derived directly from separate calculations on the fragments defining the environment. This allows modeling of a wide variety of molecular environments without the need for any predefined parameters, whereas the QM/FQ and QM/FQFμ models have mainly been applied to aqueous environments. Second, although we limit ourselves to molecular gradients and Hessians in this work, the implementation is introduced in the open-ended framework of OpenRSP as a first step in the direction of computing general property derivatives as well as higher-order derivatives. This will enable simulations of a great number of different spectroscopic techniques on molecules embedded in atomistic environments.

The implementation is demonstrated through proof-of-principle calculations on acetone in various solvents. Acetone was selected as the model system because it has a rather simple vibrational spectrum and its semipolar nature makes it soluble in both polar and nonpolar solvents.

In the following, we first present the key quantities needed to calculate the additional contributions from the PE model to the molecular Hessian and the dipole and polarizability gradients in Section 2. In Section 3, we provide the computational details before we present our results in Section 4 for the computed IR and Raman spectra. We end the paper in Section 5 with some concluding remarks.

## 2 Theory

In this section, we first present a brief summary of the theory for IR and Raman spectroscopy in the double-harmonic approximation. Within this approximation, the necessary components are the molecular Hessian and first-order derivatives of the dipole moment and polarizability with respect to nuclear displacements. The calculation of these properties at quantum-mechanical levels of theory is well-established for molecular systems *in vacuo*. The reader is referred to the relevant literature for details, see, for example, ref (6). Here, we focus on the contributions that arise when a molecule is embedded in a polarizable environment—specifically, when this environment is described using the PE model. The theoretical foundation for the PE model and its formulation within quantum-mechanical response theory has been extensively covered in earlier works.^47,48,62,66−68^ Here, we present the basic equations of the PE model expressed in an open-ended form in terms of the order of the multipoles and polarizabilities in the environment. While the expressions are general, in the present work, we use the standard PE potential, which is limited to permanent multipoles up to and including quadrupoles and dipole–dipole polarizabilities. The use of higher-order polarizabilities will be explored in a future study. In the last part of this section, we present the contributions from the PE model to the second-order geometric derivatives of the energy as well as to the first-order geometric derivatives of the dipole moment and polarizability. The equations will be expressed in an AO SCF formulation, following earlier works.^58,59,62^

### 2.1 Vibrational Frequencies and IR/Raman Intensities

The harmonic approximation is frequently employed when describing vibrational wave functions and builds on a Taylor expansion of the energy *E* in terms of a set of mass-weighted nuclear Cartesian displacement coordinates relative to the equilibrium geometry
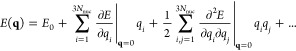
1

The displacement coordinates are given by
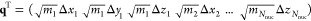
2where *m*_*n*_ is the mass of nucleus *n* and Δ*x*_*n*_, Δ*y*_*n*_, and Δ*z*_*n*_ are the nuclear displacements from the equilibrium geometry of the Cartesian coordinates of nucleus *n*. The sums in eq 1 thus run over all Cartesian coordinates of the molecular geometry and the subscript **q** = 0 denotes that the derivatives are evaluated at the equilibrium geometry. The first term on the right-hand side of the equation is the energy at the equilibrium geometry that does not depend on the displacement coordinates and is therefore not important in the further analysis in this work. The second term contains the mass-weighted molecular gradient ∂*E*/∂*q*_*i*_, which is zero at the equilibrium geometry, and the last term contains the mass-weighted molecular Hessian ∂^2^*E*/∂*q*_*i*_∂*q*_*j*_. Through an eigenanalysis of the Hessian,^5^ one can obtain the normal-mode frequencies from the eigenvalues, whereas the eigenvectors correspond to a transformation matrix that defines each normal coordinate *Q*_*I*_ in terms of Cartesian displacements. Three of the eigenvectors correspond to the overall translation of the system and three eigenvectors (two for linear molecules) correspond to the overall rotation of the system.

IR intensities are often reported in terms of the *molar decadic attenuation coefficient*, ε, which has units m^2^·mol^–1^. To facilitate comparisons to other works, we summarize the commonly used units for reporting IR intensities in Table 1. Within the double-harmonic approximation, ε for vibrational mode *I* is obtained from the equation
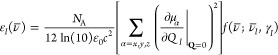
3where *N*_A_ is the Avogadro constant, *c* is the speed of light, ε_0_ is the vacuum permittivity, and μ_α_ is a Cartesian component of the electric dipole moment. The lineshape function *f*(ν̅; ν̅_*I*_, γ_*I*_) is introduced to take into account homogeneous broadening effects, such as the finite lifetime of the excited vibrational states. In this work, we use a Cauchy distribution with a damping factor γ_*I*_ so that^6^
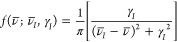
4where ν̅ is the wavenumber of the incident radiation and ν̅_*I*_ is the wavenumber associated with vibrational mode *I*. The lineshape function broadens the peaks with a half-width at half-maximum (HWHM) of the peak associated with mode *I* being γ_*I*_. The dipole moment gradient can also be expressed as a mixed energy derivative
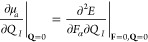
5where *F*_α_ is the αth component of the electric-field strength and the derivative is evaluated at zero-field strength and at the equilibrium geometry.

**Table 1:** Units of the Most Commonly Reported IR Intensities^6,69^

property	unit	origin
	C^2^·kg^–1^	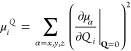
	D^2^·Å^–2^·amu^–1^	1.4924 × 1012·μ_*i*_^*Q*^
molar decadic attenuation coefficient	m^2^·mol^–1^	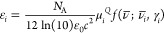
molar decadic attenuation coefficient	L·mol^–1^·cm^–1^	10·ε_*i*_
Napierian integrated molecular attenuation coefficient	m·mol^–1^	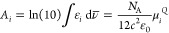
Napierian integrated molecular attenuation coefficient	km·mol^–1^	10^–3^·*A*_*i*_

Just as for IR, there are several commonly used ways to report Raman intensities, but most are related to the *absolute differential Raman scattering cross section*, σ′, with units C^4^·s^2^·J^–1^·m^–2^·kg^–1^. Some of the most commonly used Raman units are reported in Table 2. In the double-harmonic approximation and at temperature *T*, σ′ is computed as^70,71^
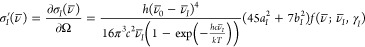
6where σ is the total scattering cross section, Ω is the solid angle, ν̅_0_ is the wavenumber of the incident light, and *k* is the Boltzmann constant. The constants 45 and 7 stem from the fact that we evaluate σ′ for an experimental setup where the light entering the sample is polarized perpendicular to the direction of observation and its propagation.^5^ Other choices of combination coefficients belong to other experimental setups. The Raman invariants *a*_*I*_ and *b*_*I*_^2^ are given by^72^
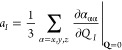
7and
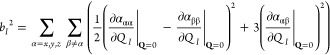
8respectively, where α_αβ_ is the component of the electric dipole–dipole polarizability corresponding to Cartesian coordinates α and β. As the frequency-dependent polarizability gradient involves a frequency-dependent electric field, it cannot be directly represented as an energy derivative. Instead, a quasi-energy, *Ẽ*, (which reduces to the energy in the absence of a frequency-dependent electric field) derivative is used^4,6,73,74^
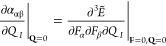
9where the derivative is evaluated at zero-field strengths and at the equilibrium geometry.

**Table 2:** Units of the Most Commonly Reported Raman Intensities^70,75^

property	unit	origin^a^
	C^4^·m^2^·J^–2^·kg^–1^	α_*i*_^*Q*^ = *k*_*a*_*a*_*i*_^2^ + *k*_*b*_*b*_*i*_^2^
	m^4^·kg^–1^	(1/4πε_0_)^2^α_*i*_^*Q*^
	Å^4^·amu^–1^	1.3413 × 10^33^·α_*i*_^Q^
absolute differential scattering cross section	C^4^·s^2^·J^–1^·m^–2^·kg^–1^	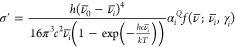

a Values of combination coefficients *k*_*a*_ and *k*_*b*_ depend on the experimental setup.^5^ We use *k*_*a*_ = 45 and *k*_*b*_ = 7 in the present work.

### 2.2 Polarizable Embedding

The PE model is an atomistic classical scheme for efficiently and accurately including complex environments in quantum-mechanical calculations. The total system is split into a core quantum region, which is described by a quantum-mechanical method, and its environment, whose effects on the core part are described effectively through an embedding potential. The environment is further partitioned into computationally manageable fragments. In the case of solvents, the fragments typically consist of individual solvent molecules, while a fragmentation procedure is used for more complex environments.^50,67^ For each fragment, a quantum-mechanical calculation is performed, producing a set of electric multipoles and polarizabilities that are distributed to a number of sites within the fragment, usually the atomic centers. Alternatively, the multipoles and polarizabilities can be taken from existing preparametrized potentials that have been derived for proteins,^57^ a series of solvents,^55^ and a few lipids.^76^

The energy of a quantum region in the presence of an environment can be separated into two contributions
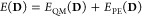
10where *E*_QM_(**D**) is the energy of the quantum region, *E*_PE_(**D**) is the embedding energy that describes the interactions between the quantum region and the environment, and **D** is the AO density matrix. In this work, Kohn–Sham DFT (KS-DFT) is used for the quantum region, thus *E*_QM_(**D**) = *E*_DFT_(**D**). The KS-DFT energy is given by
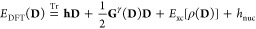
11where  indicates that the trace is taken of each term on the right-hand side, **h** contains the one-electron terms (kinetic energy and electron–nuclear attraction), **G**^γ^(**D**) contains the two-electron terms (electronic Coulomb and fractional exchange interactions), *E*_xc_[ρ(**D**)] is the exchange–correlation contribution as a (nonlinear) functional of the density, and *h*_nuc_ is the nuclear–nuclear interaction energy. We will not go into further details about these terms here, but we note that the dependence of the individual contributions to the energy on the AO density matrix is either independent (*h*_nuc_), linear (**hD**), quadratic (**G**(**D**)**D**), or nontrivial (*E*_xc_[ρ(**D**)]), and that this separation of terms into orders of density-matrix dependence is used by OpenRSP. The contributions from the PE model to be presented in the following can also be grouped into zeroth-, first-, and second-order density-matrix dependence. We have chosen to do so in this work to align our implementation with the corresponding interfaces to OpenRSP.

The PE energy can be written as
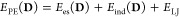
12where *E*_es_(**D**) is the electrostatic energy from the interaction between the permanent multipoles in the environment and the electrons and nuclei in the quantum region, *E*_ind_(**D**) is the induction energy resulting from the polarization of the environment modeled by induced multipoles, and *E*_LJ_ is the energy due to nonelectrostatic repulsion and dispersion interactions modeled by a 6-12 Lennard-Jones (LJ) potential.

In the following, we will present each of the energy contributions. For the electrostatic and induction energies, we will make use of a multi-index notation^77^ that allows us to write compact expressions that are open-ended in terms of the order of the multipoles and polarizabilities. A multi-index is denoted by α, β, and so forth and consists of three indices associated with the three Cartesian coordinates [*i.e.*, α = (α_*x*_, α_*y*_, α_*z*_)]. The addition and subtraction of multi-indices is performed component-wise, that is, α ± β = (α_*x*_ ± β_*x*_, α_*y*_ ± β_*y*_, α_*z*_ ± β_*z*_). The absolute value of a multi-index is defined as |α| = α_*x*_ + α_*y*_ + α_*z*_, and the factorial as α! = α_*x*_!α_*y*_!α_*z*_! The multi-index power is given by **R**^α^ = *R*^α_x_^*R*^α_*y*_^*R*^α_*z*_^. A partial derivative is written as . Summing over the absolute value of a multi-index implicitly includes a sum over all possible multi-indices for each of the absolute values in the sum, for example, ∑_|α|=0_^1^α = (0, 0, 0) + (1, 0, 0) + (0, 1, 0) + (0, 0, 1). A Cartesian component of a tensor is specified with a multi-index in square brackets, for example, *T*^[α]^.

The electrostatic energy describes the interactions between the electrons and nuclei in the quantum region and the permanent multipoles in the environment. Using the multi-index notation, we can write it as
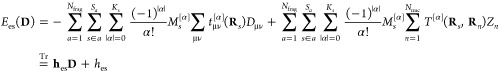
13where *N*_frag_ is the number of fragments in the environment, *S*_*a*_ is the number of sites in fragment *a*, *K*_*s*_ is the maximum order of the multipoles on site *s* in fragment *a*, *M*_*s*_^[α]^ is a component of a Cartesian multipole on site *s*, μ and ν are indices of the AOs belonging to the quantum part, *t*_μν_^[α]^(**R**_*s*_) is the μνth element of the |α|th-order derivative of a one-electron electrostatic-potential integral, *D*_μν_ is the μνth element of the AO density matrix, *T*^[α]^(**R**_*s*_, **R**_*n*_) is a component of a Cartesian interaction tensor involving the positions of site *s* and nucleus *n*, *N*_nuc_ is the number of nuclei in the quantum region, and *Z*_*n*_ is the charge of nucleus *n*. An interaction tensor is generally defined as
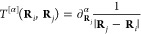
14where the subscript on the multi-index partial-derivative operator denotes the coordinate that the derivative is taken with respect to. The *t*_μν_^[α]^(**R**_*s*_) integrals can be defined in terms of interaction tensors as
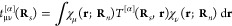
15where **r** is the electron coordinate and χ_μ_(**r**; **R**_*n*_) and χ_ν_(**r**; **R**_*n*_) are AOs with a parametric dependence on the nuclear coordinates. The multipole–electron part of the interaction energy depends linearly on the density matrix while the multipole–nuclear interaction is a scalar that does not depend on the density matrix, as shown in the last equality of eq 13.

The second energy term in eq 12 is the induction energy, which is the result of the polarization of the environment. The polarization is modeled using polarizabilities that give rise to induced multipoles describing the response of a given fragment to the fields from the electrons and nuclei in the quantum part as well as the permanent multipoles in the environment. The induction energy can be formulated in terms of a generalized classical linear-response matrix of Cartesian polytensors (which are defined as a set of Cartesian tensors in a sequence of increasing rank)^78^
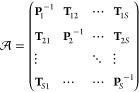
16whose diagonal blocks contain inverse Cartesian polytensors that themselves consist of the multipole–multipole polarizabilities of a given site while the off-diagonal blocks hold the corresponding polytensors that consist of interaction tensors which describe the interaction between polarizable sites. The induction energy can then be written as
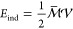
17where  is a matrix containing polytensors of the induced multipoles and  is a matrix that consists of polytensors that contain the derivatives of the electrostatic potential from the electrons, nuclei, and permanent multipoles at the polarizable sites. The induced multipoles can be determined by solving the matrix equation
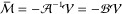
18

In practice, the matrix equation is never solved explicitly, since the linear-response matrix quickly becomes too large for environments with many sites, and instead an iterative solver is used. Using the multi-index notation, the induction energy can be written as
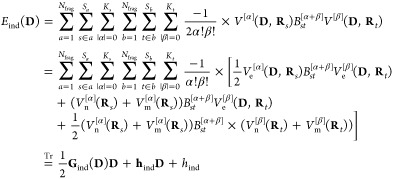
19where *V*^[α]^(**D**, **R**_*s*_) is a component of the |α|th-order derivative of the electrostatic potential and *B*_*st*_^[α+β]^ is a component of the *st*th block of the inverse of the linear-response matrix in eq 16. In the second equality, we expand the energy in terms of derivatives of the electrostatic potentials from the electrons
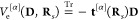
20nuclei
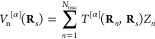
21and permanent multipoles
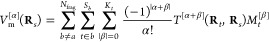
22and collect terms that depend on the density to second-, first-, and zeroth-order, respectively, which are then given in matrix form in the last equality. The sum over fragments in the multipole electrostatic potential (eq 22) excludes the fragment that contains site *s*, here assumed to be fragment *a*.

Finally, the last term in eq 12 is the LJ potential energy, which effectively describes nonelectrostatic repulsion and dispersion. It is given by
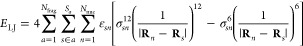
23where Lorentz–Berthelot rules are used to combine parameters, that is, σ_*sn*_ = 1/2(σ_*s*_ + σ_*n*_) and . Here, σ_*s*_ and ε_*s*_ are LJ parameters of atoms in the environment and σ_*n*_ and ε_*n*_ are LJ parameters of the atoms in the quantum region. The LJ potential energy is thus purely classical and independent of the density matrix.

The PE energy gives rise to Fock-matrix contributions that are found by minimizing the energy with respect to variations of the electron density
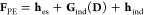
24

### 2.3 Derivatives of the PE Energy

In this section, we present the additional contributions to the geometric derivatives of the energy, dipole, and polarizability that arise for a molecule embedded in a polarizable environment described using the PE model. These, and all other contributions, that is, those for a molecule in vacuum, are considered in the framework of a density-matrix-based quasi-energy formulation (see, e.g., works by Thorvaldsen et al.^58^ and Ringholm, Jonsson, and Ruud^59^ for details). In this approach, properties are determined as derivatives of the quasi-energy Lagrangian, which up to third-order can be written as^58^
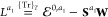
25
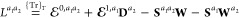
26
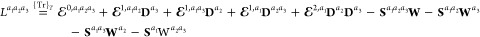
27where  means that a trace and time-average of each term on the right-hand side is taken,  is the quasi-energy, **D** is the density matrix, **S** is the overlap matrix, and **W** is the energy-weighted density matrix
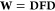
28The superscripts *a*_1_, *a*_2_, and *a*_3_ denote derivatives with respect to given perturbations (either geometric or electric dipole perturbations in this work) with associated frequencies ω_*a*_1__, ω_*a*_2__, and ω_*a*_3__, respectively. The notation employed here for quasi-energy derivatives of *n*th order is defined as
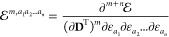
29where ε_*a*_1__, ε_*a*_2__, ..., ε_*a*_*n*__ are the strengths associated with perturbations *a*_1_, *a*_2_, ..., *a*_*n*_, respectively. The quasi-energy derivatives are expressed using the *n* + 1 rule where only *n*th-order derivatives of the density matrix are needed to calculate a property of order *n* + 1. Derivatives of the density matrix with respect to the perturbation designated as *a*_1_ are not present as a consequence of the application of the time-averaged Hellmann–Feynman theorem in the derivation of the quasi-energy gradient (eq 25). We again refer to Thorvaldsen et al.^58^ for further details concerning the approach. Finally, we note that the quasi-energy derivatives reduce to standard energy derivatives for time-independent properties.

In the following, we use superscripts *g*_1_ and *g*_2_ to denote a derivative with respect to a Cartesian component of a nuclear coordinate, and superscripts *f*_1_ and *f*_2_ to denote a derivative with respect to a Cartesian component of the external field. For the molecular properties treated in this work, the contributions from the interactions between the quantum region and its environment are found by taking the relevant derivatives of the interaction energies according to the forms indicated in eqs 25–27. These expressions contain perturbed density and Fock matrices, the latter as part of the perturbed energy-weighted density matrix. The calculation of perturbed density and Fock matrices also entails the evaluation of contributions stemming from the derivatives of the PE Fock matrix (eq 24) and contributions to the electronic Hessian when solving the response equations. We refer to previous work^58,59^ for details about the general method used to obtain perturbed density and Fock matrices. We note, however, that the additional contributions to the perturbed density and Fock matrices arising from the interaction between the quantum region and its environment are included in the following presentation, although only derivatives of the energy are explicitly addressed.

We begin with the derivatives of the electrostatic interaction energy (eq 13). Here, there is a dependence on nuclear positions in the nuclear–multipole part and through the AOs in the electrostatic-potential integrals (eq 15) that appear in the electron–multipole part. The contributions from the electrostatic interactions to the geometric gradient and Hessian are
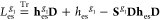
30
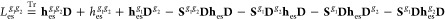
31and the contributions to the dipole and polarizability gradients are
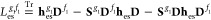
32
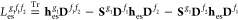
33

Since we do not consider local field effects in this work, **h**_es_ and *h*_es_ are independent of the external field. The first- and second-order geometric derivatives of the **h**_es_ matrix that appear in eqs 30–33 are given by
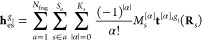
34
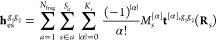
35and the derivatives of *h*_es_ are
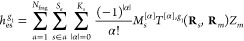
36
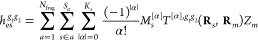
37

In the last two equations, **R**_*m*_ and *Z*_*m*_ are the coordinate and charge, respectively, of nucleus *m* which is the only one that remains after the differentiation of *T*^[α]^ with respect to *g*_1_ for the first-order derivative and to *g*_1_ and *g*_2_ for the second-order derivative.

We next consider the induction energy (eq 19) where there is a dependence on nuclear positions through the nuclear and electronic electrostatic potentials. The contributions to the geometric gradient and Hessian from the induction energy are given by
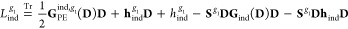
38
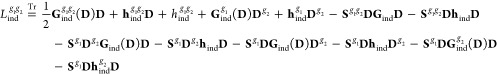
39and the contributions to the dipole and polarizability gradients are given by
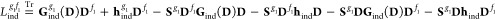
40
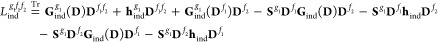
41

Inserting the expression for the electronic electrostatic potential (eq 20) allows us to write the first- and second-order geometric derivatives of the **G**_ind_(**D**) matrix as
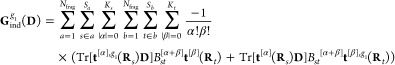
42
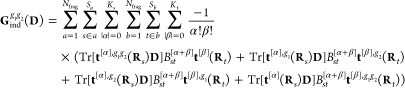
43
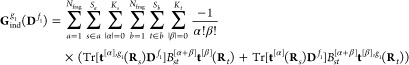
44

The first- and second-order derivatives of the **h**_ind_ matrix are given by
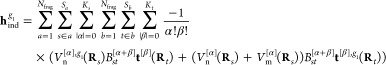
45
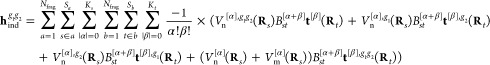
46

The electrostatic potentials from the nuclei and multipoles that appear here are defined in eqs 21 and 22, respectively. Geometric derivatives of the electrostatic potentials from the multipoles disappear as they do not depend on nuclear positions, while the derivatives of the nuclear electrostatic potential are given by
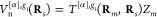
47
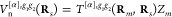
48

The last part of the geometric derivatives of the induction energy is *h*_ind_, which depends on nuclear positions through the nuclear electrostatic potential. The first- and second-order derivatives of this term are
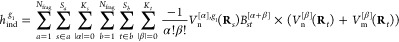
49
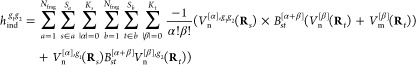
50where the geometric derivatives of the nuclear electrostatic potential are given in eqs 47 and 48.

Finally, there is the LJ potential energy (eq 23) that only contributes to the geometric gradient and Hessian because it neither depends on the external field nor the density matrix. The contributions to the geometric gradient and Hessian are
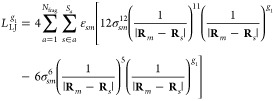
51
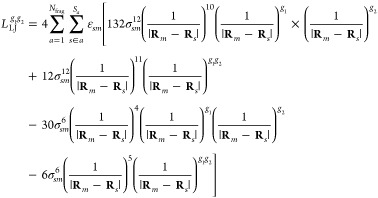
52where subscript *m* refers to the nucleus that survives the differentiation with respect to *g*_1_ for the first-order derivative and to *g*_1_ and *g*_2_ for the second-order derivative.

## 3 Computational Details

The properties needed to simulate harmonic IR and Raman spectra were calculated for acetone in three different solvents, namely, water, chloroform, and acetone. To simulate IR and Raman spectra of solute–solvent systems, it is necessary to adequately sample the configurational space. In this work, we sampled structures for all three systems from classical MD simulations. The partial Hessian and first-order dipole and polarizability derivatives were then calculated for each structure. The final spectra were obtained by convolution of the spectra of each structure. Spectra of acetone in vacuum and acetone in the three solvents using the PCM were also computed for comparison. In the following, we provide the details for each step. All input and output files, as well as scripts used to run the calculations and to extract data, have been deposited on Zenodo.^79^

### 3.1 Generation of Structures

Classical MD simulations were performed using GROMACS 2019.3^80−82^ employing the OPLS-AA force field.^83^ The OPLS-AA topologies for acetone and chloroform were taken from the GROMACS molecule and liquid database at virtualchemistry.org.^84,85^ The TIP3P potential^86^ was used for water. Initial cubic boxes of 60 × 60 × 60 Å were created and the system was then minimized with 100 steps of steepest descent and 1000 steps of conjugate gradient (5000 in the case of chloroform solvent). An equilibration protocol containing both *NPT* and *NVT* ensembles was performed. For water and acetone solvents, an initial simulation in the *NPT* ensemble was run for 0.5 ns, followed by a 2 ns simulation in the *NVT* ensemble. Because of difficulties with the equilibration of acetone in chloroform (see ref (79) for details), these two steps were preceded by two additional equilibration steps, consisting of a 0.0001 ps *NVT* simulation and a 0.05 ps *NPT* simulation, with time steps of 0.01 and 0.1 fs, respectively. Initial velocities were taken from a Maxwell distribution at 298 K. All simulations were performed with periodic boundary conditions, the leap-frog integrator, and a time step of 1 fs (for all except the aforementioned additional steps for acetone in chloroform). Nonbonded interactions were cut off at 15 Å and electrostatic interactions beyond the cutoff were treated using the smooth particle-mesh Ewald^87^ method. The Berendsen thermostat (298 K) and barostat (1 bar) were used with a coupling constant of 0.5 ps to maintain the temperature and pressure in the *NPT* equilibration.^88^ The velocity-rescaling thermostat^89^ with a coupling constant of 0.5 ps was used to maintain the temperature at 298 K in the *NVT* simulations. After the equilibration steps, a 10 ns *NVT* production run was performed. We then extracted 250 snapshots at 10 ps intervals from the first 2.5 ns of the final trajectory.

### 3.2 Geometry Optimization and Property Calculations

**Figure A1:**
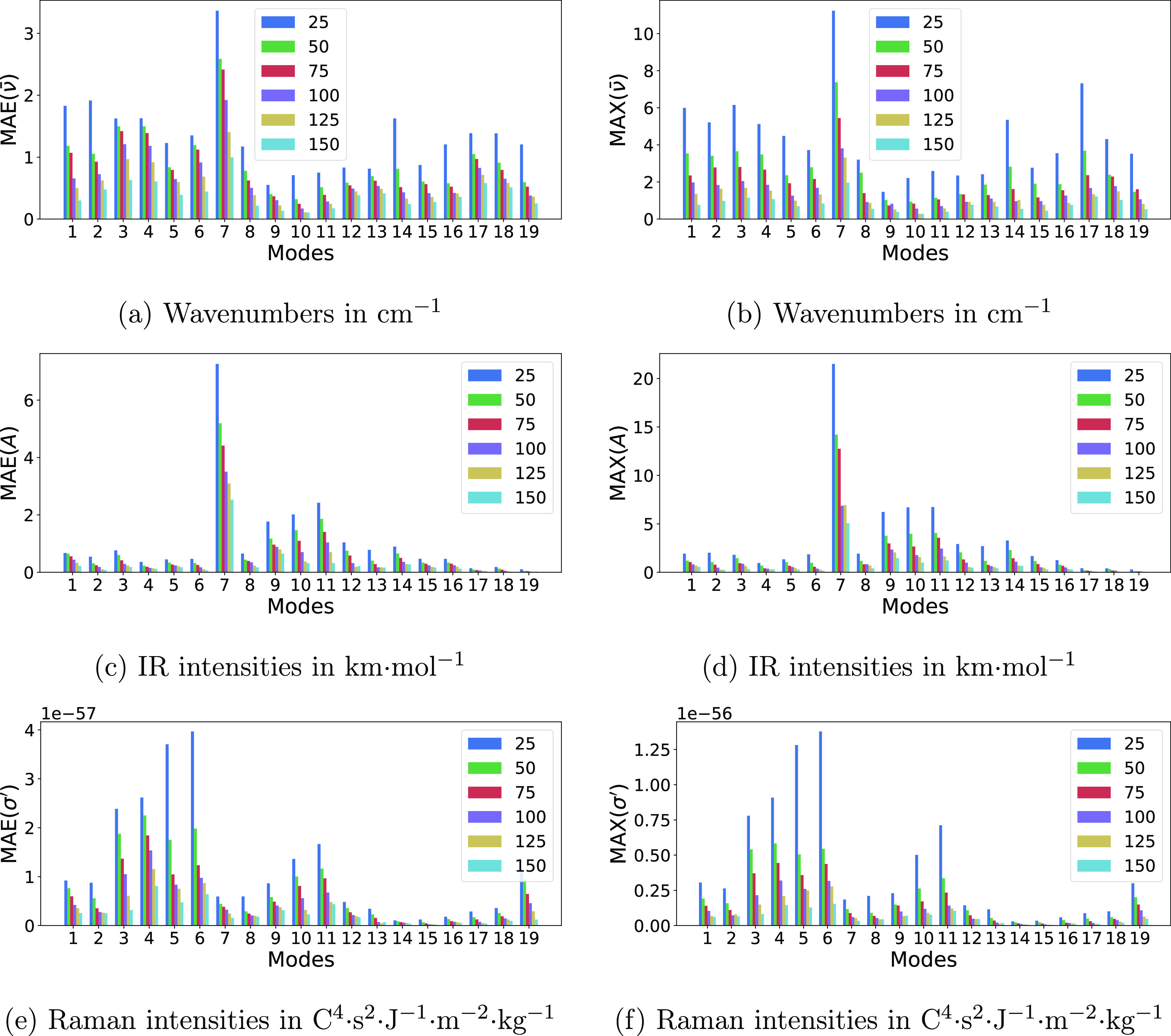
Convergence with respect to the sample size. Plots show MAE and MAX of moving averages of a given sample size for all vibrational modes above 750 cm^–1^ compared to a sample size of 250. All calculations were performed using HF/pcseg-1 with acetone embedded in a 12 Å water shell.

For each structure extracted from the MD trajectory, the geometry of the central acetone molecule was first optimized in the presence of rigid solvent molecules. The partial Hessian and first-order dipole and polarizability derivatives were then calculated using the optimized structures. The 250 equidistant snapshots that were extracted from the MD trajectory were used to perform a preliminary analysis of the convergence with respect to sample size (see Section A.1). Based on this analysis, and taking into account the computational cost, we found that 50 equidistant snapshots is adequate for our purposes, which is to demonstrate our implementation through proof-of-principle calculations. We note here that with 50 snapshots, we could produce Raman spectra that are well-converged with respect to the number of snapshots, whereas comparatively larger errors were observed for some IR-active modes, in particular, the carbonyl stretching mode (see Figure A1).

The effects from the solvent were modeled by embedding potentials produced using PyFraME.^90^ The solvent was extracted using a center-of-mass distance criterion, that is, solvent molecules with their center of mass within the cutoff distance from the center of mass of the central acetone were included. We used a cutoff distance of 12 Å which results in adequate accuracy (see Section A.2). For each solvent molecule in the solvent shell, atom-centered multipoles up to and including quadrupoles and atom-centered dipole–dipole polarizabilities were derived using the LoProp scheme.^91,92^ For this, a calculation using the Dalton program^93,94^ is performed employing the B3LYP^95−99^ exchange–correlation functional and a recontracted version of 6-31+G*^100−102^ (called loprop-6-31+G* in Dalton). LJ parameters were taken from the OPLS-AA force field.

All geometry optimizations were performed at the PBE0^103−106^/pcseg-2^107^ level of theory. The PBE0 functional was chosen based on its accuracy in the modeling of molecular geometries.^105^ The pcseg-2 basis set was chosen as it has been shown to give good results with DFT for both molecular structures and vibrational properties.^108^ Additional support for the choice of a triple-ζ basis was found through a convergence analysis that showed it to be a good compromise between accuracy and computational cost compared to its double- and quadruple-ζ counterparts (see Section A.3). The LSDalton program^75,93^ was used for optimizations in vacuum and in solvent utilizing the FraME library^109^ for the environment contributions. These optimizations used an initial numerical Hessian and Baker convergence criteria.^110^ The default exchange–correlation integration grid was used but with a radial integration accuracy of 2.154 × 10^–17^ and an angular expansion order of 47, which corresponds to 60 radial points for second-row atoms and up to 770 angular points (adjusted down by pruning near the nuclei). A few snapshots were discarded at this stage due to convergence issues. Therefore 49, 48, and 47 snapshots for water, acetone, and chloroform solvents, respectively, were used in the subsequent property calculations. Geometry optimizations utilizing the PCM were performed with Gaussian 16^111^ using the pcseg-2 basis set obtained from the Basis Set Exchange.^112^ The geometry optimizations using Gaussian were performed with a tight SCF threshold (SCF=VeryTight) and a fine integration grid (Int=SuperFine). To accompany the PCM-based structures, we also performed a geometry optimization in vacuum using Gaussian with the same settings.

The partial Hessian and first-order dipole and polarizability derivatives of acetone in vacuum and in environments described using the PE model were calculated using LSDalton, FraME, and OpenRSP.^58,59,64^ Gaussian was used for the PCM-based calculations and its accompanying vacuum calculations. The same settings were used for the property calculations as for the geometry optimizations. As can be seen from Tables S4 and S8 in the Supporting Information, there are small differences between the frequencies and intensities obtained using LSDalton and those obtained using Gaussian. This does not affect the discussion of the results since the focus is on solvent effects and vacuum to solvent shifts are calculated consistently. The frequency-dependent polarizability derivatives were calculated using an input wavelength of 514.5 nm. This corresponds to an argon laser that has been used in Raman experiments on aqueous acetone.^113^ The energy derivatives and molecular geometry were used by the vibrational spectroscopy package SpectroscPy^114^ to perform a Hessian eigenvalue analysis to obtain the harmonic vibrational frequencies and normal coordinates and to calculate the IR and Raman intensities. Raman intensities were calculated at 298 K. IR and Raman spectra were generated by combining the individual spectra of each structure into a single spectrum. Specifically, for IR, we use
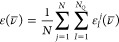
53where *N* is the number of snapshots, *N*_*Q*_ is the number of vibrational modes, and ε_*I*_^*j*^(ν̅) is the molar decadic attenuation coefficient of the *I*th vibrational mode in snapshot *j* (eq 3). For Raman, we similarly use
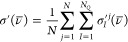
54where σ_*I*_^′*j*^(ν̅) is the absolute differential scattering cross section of the *I*th vibrational mode in snapshot *j* (eq 6). The Cauchy distribution was used in both the IR and Raman cases as a basis for a lineshape function with an HWHM of 3.0 cm^–1^ for all modes (see eq 4).

As mentioned in Section 2.1, the harmonic vibrational frequencies are found from an eigenanalysis of the molecular Hessian in mass-weighted Cartesian coordinates. This produces 3*N* frequencies and the corresponding normal modes, but not all of these are vibrational, as six of these (five for linear molecules) describe an overall translation and rotation of the molecule. In order to distinguish between low-frequency vibrational modes and the translational and rotational modes, it is common to project out translation and rotation from the Hessian. However, this approach cannot be used here, since we use the PHVA approximation. Moreover, the core molecule is embedded in a rigid solvent cage and is therefore no longer free to move around in space. This will inevitably introduce errors in our calculations. Low-frequency modes are especially susceptible to contamination by translational and rotational motions. Visual inspection of an arbitrarily chosen snapshot indicated that the six modes with the lowest energy do not correspond to purely translational and rotational motion and also that additional low-frequency modes show some extent of global motion. Simply removing the six modes of the lowest frequency is therefore not a good choice for the embedded systems. Instead, we identified from the visual inspection a cutoff at 750 cm^–1^ above which the modes have only negligible contamination of translational and rotational motion. We do not consider or discuss the normal modes with lower frequencies due to these impurities. By comparing the frequencies obtained with and without projecting out translation and rotation, we estimate that the error in the remaining vibrational modes is only a few cm^–1^ for the localized higher-frequency modes and never exceeds 10 cm^–1^ on average for any mode (see Figure S1 in the Supporting Information).

## 4 Results and Discussion

**Figure 1:**
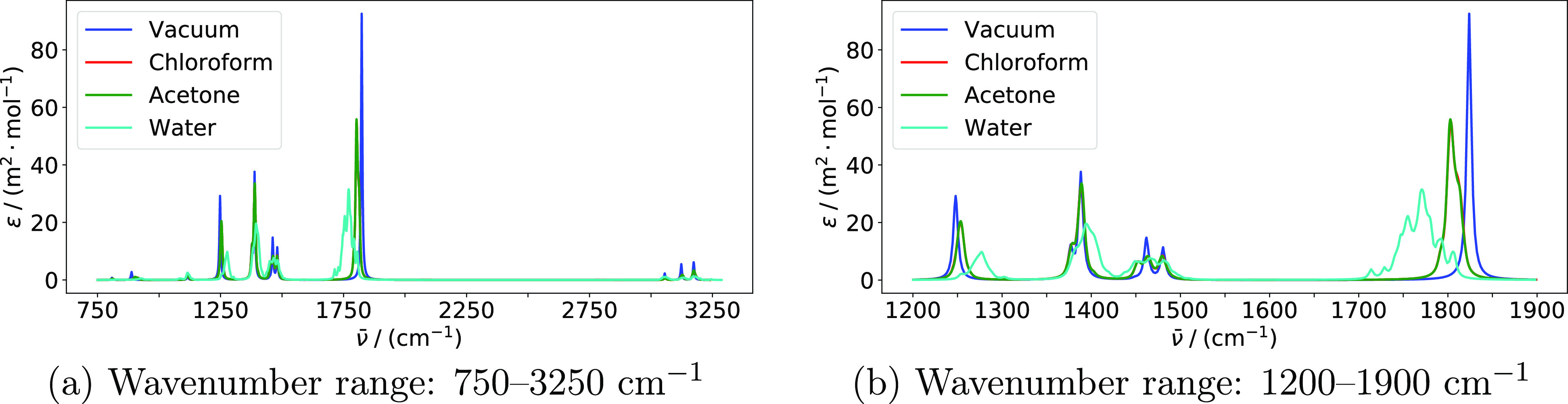
IR spectra of acetone in various environments modeled using the PE model. Spectra are based on averages over all snapshots. Calculations were performed using PE-PBE0/pcseg-2 with acetone embedded in a 12 Å solvent shell. An HWHM value of 3.0 cm^–1^ was used to broaden individual peaks. Only modes above 750 cm^–1^ are included. Panel (b) displays the part of the spectrum with the highest IR absorption.

**Figure 2:**
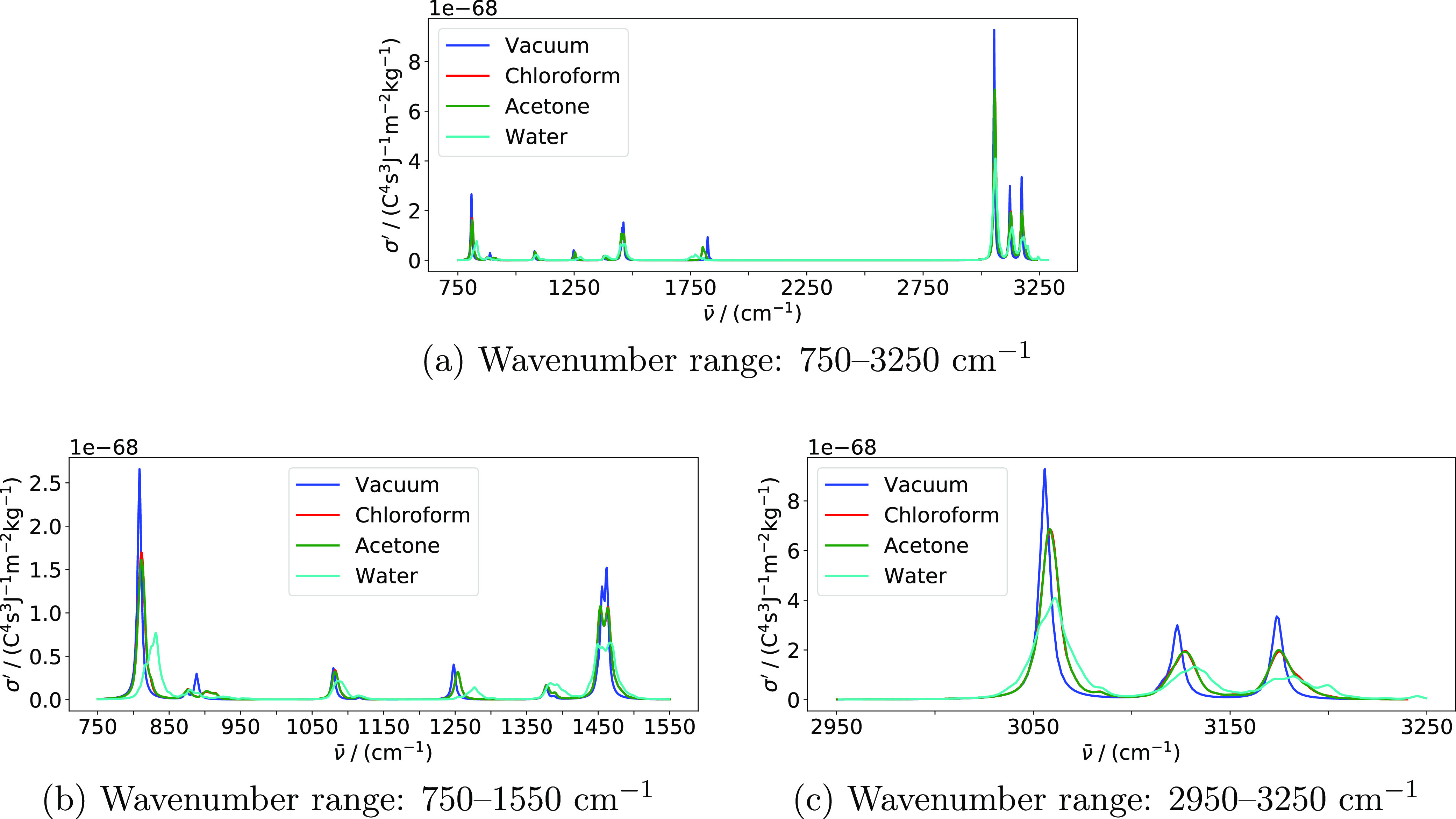
Raman spectra of acetone in various environments modeled using the PE model. Spectra are based on averages over all snapshots. Calculations were performed using PE-PBE0/pcseg-2 with acetone embedded in a 12 Å solvent shell and using an input wavelength of 514.5 nm. An HWHM value of 3.0 cm^–1^ was used to broaden individual peaks. Only modes above 750 cm^–1^ are included. Panels (b,c) display the parts of the spectrum with the highest Raman activity.

Harmonic IR and Raman spectra of acetone in vacuum and in water, chloroform, and acetone solutions are presented in Figures 1 and 2, respectively. Averaged vibrational wavenumbers and associated IR and Raman intensities are tabulated in the Supporting Information. Our focus in the discussion is on the inclusion of the effect of different solvents through the PE model and PCM. In addition, we present convergence tests with respect to basis set, environment size, and sampling size in the Appendix. We do, however, point out that there are several other factors that influence the accuracy of the calculations that are beyond the scope of the present work, including the quantum-mechanical level of theory and the fact that we do not consider anharmonic effects.

The three strongest peaks in the IR spectra presented in Figure 1 can be assigned^115^ to the carbonyl stretching mode (around 1800 cm^–1^), the symmetric methyl deformation (umbrella) mode (around 1390 cm^–1^), and the asymmetric C–C stretching mode (around 1260 cm^–1^). We will limit the following discussion to these three peaks. It is worth noting that the spectra for acetone in chloroform and acetone solutions are virtually overlapping, suggesting no significant differences in the solute–solvent structure and dynamics for these two solvents. The effect of hydrogen bonding between the acetone solute and water solvent is evident from the −53 cm^–1^ shift of the carbonyl stretching mode relative to vacuum, whereas the shift is −21 cm^–1^ in chloroform and acetone solvents. The C–C stretching mode is shifted to higher wavenumbers by the solvents, although less in magnitude. Indeed, this shift is +29 cm^–1^ in water and only +6 cm^–1^ in chloroform and acetone. The wavenumber of the methyl umbrella mode is only slightly shifted by the water solvent (+6 cm^–1^) while it is unaffected by the chloroform and acetone solvents. These shifts correlate well with the change in the bond lengths that are presented in Table 3.

**Table 3:** Bond Lengths (in angstrom) of Acetone in Vacuum and Different Solvents Modeled Using the PE Model and the PCM

		chloroform		acetone		water	
bond	vacuum	PE^a^	PCM	PE^a^	PCM	PE^a^	PCM
C=O	1.206	1.210 (0.001)	1.211	1.210 (0.001)	1.213	1.221 (0.005)	1.213
C—C	1.507	1.503 (0.002)	1.503	1.503 (0.002)	1.502	1.493 (0.006)	1.501
C—H	1.093	1.091 (0.003)	1.093	1.091 (0.003)	1.092	1.091 (0.003)	1.092
C—H′	1.087	1.091 (0.003)	1.087	1.091 (0.003)	1.087	1.091 (0.003)	1.087

a Average over all snapshots with standard deviations in parentheses.

Acetone in aqueous solution forms hydrogen bonds with two water molecules on average, which results in an elongation of the carbonyl bond and a subsequent shift of the carbonyl stretch to lower wavenumbers. The C–C bonds, on the other hand, are contracted, which results in a shift of the C–C stretching frequency to higher wavenumbers. The methyl umbrella mode can be linked to the H–C–C bond angles, which vary only slightly in the presence of a solvent and are always between 109 and 111°.

The configurational variety in the snapshots extracted from the MD simulation causes an inhomogeneous broadening. Even though the broadening of the peaks in the spectrum is in part determined by the chosen broadening factor, a comparison between the different solvents can be made. The most substantial broadening in the IR spectrum (Figure 1) is observed for the carbonyl stretch in water. Correspondingly, the standard deviations associated with the calculated wavenumber and IR intensity are 20 cm^–1^ and 23 km·mol^–1^, respectively. This is roughly three times larger than the standard deviations in chloroform and acetone, which are 6 cm^–1^ and 8 km·mol^–1^ for both solvents. The broadening of the carbonyl stretching mode in water can in part be attributed to the strong hydrogen-bonding solvent. In contrast, the weaker dipole–dipole interactions between the acetone solute and chloroform and acetone solvent molecules result in smaller shifts and less-pronounced broadening. The fine structure of the carbonyl stretching peak is most likely due to limited sampling.

Raman spectra calculated with an input wavelength of 514.5 nm are shown in Figure 2. The strongest peaks in the Raman spectrum can be assigned^115^ to the symmetric and asymmetric C–H stretching modes (above 3000 cm^–1^), the symmetric C–C stretch (around 800 cm^–1^), and the asymmetric methyl deformation modes (around 1450 cm^–1^). The spectra for the chloroform and acetone solutions are overlapping also for Raman scattering. Solvent effects are most apparent by the +23 cm^–1^ shift and substantial broadening (standard deviation of 16 cm^–1^) of the C–C symmetric stretch in water. The corresponding shift in acetone and chloroform is only minor (+3 cm^–1^). This is in agreement with the shortening of the C–C bond, which is 0.014 Å in water, 0.004 Å in chloroform, and 0.005 Å in acetone (Table 3). The frequencies of the methyl deformation modes are virtually unchanged when adding a solvent. The symmetric C–H stretch is shifted by +5 cm^–1^ in water and +2 cm^–1^ in acetone and chloroform. The asymmetric C–H stretches are shifted by +9 cm^–1^ in water, +4 cm^–1^ in acetone, and +1 cm^–1^ in chloroform. The broadening of these peaks is in part due to larger separation of the two modes underlying each of the peaks. In the case of the highest-frequency band in water, however, there is also a large spread of the wavenumbers of both underlying modes, with standard deviations of 17 and 13 cm^–1^, respectively.

A question that naturally arises is whether the additional computational cost of the configurational sampling associated with the PE model is reasonable compared to using a continuum solvation model. To answer this question, we calculated IR and Raman spectra using the PCM. Before comparing the spectra, we will briefly examine the effect on the geometry of the acetone solute. We note that the differences in bond lengths (Table 3) are small and may be of the same order as numerical errors, such as those introduced by the tessellation of the molecular cavity in the PCM. The addition of a solvent through PCM also leads to slight elongation of the C=O bond and slight shortening of the C–C bond and virtually no effect on the C–H bond length. The solvent effect on acetone bond lengths in chloroform, acetone, and water is very similar using the PCM. In other words, the larger solvent shift in water found in the PE calculations is not reproduced using the PCM. This reflects the lack of specific interactions (hydrogen bonds) in the PCM.

**Figure 3:**
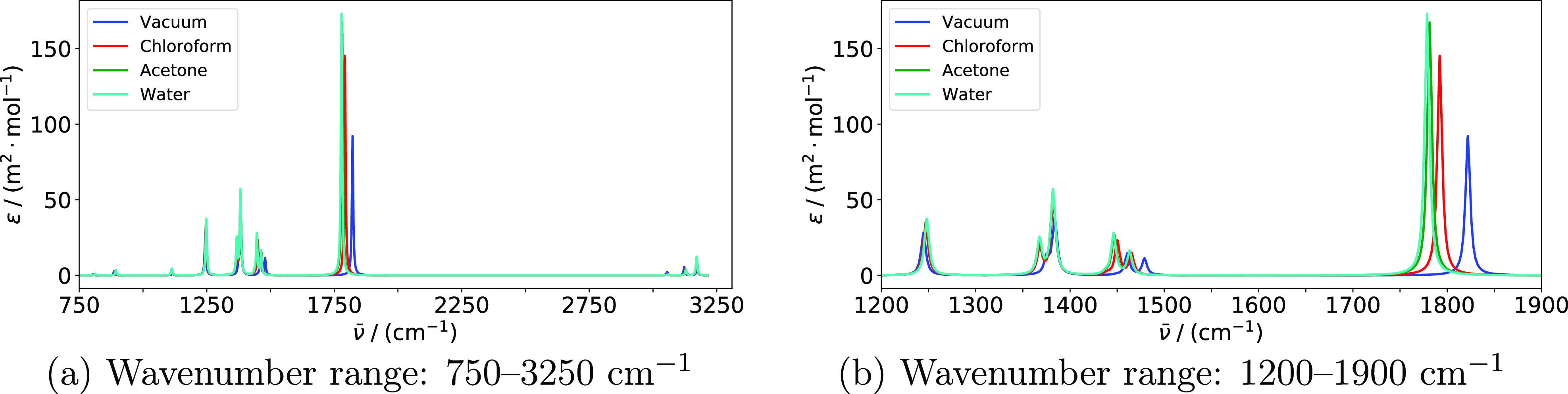
IR spectra of acetone in various environments modeled using the PCM. Calculations were performed using PCM-PBE0/pcseg-2. An HWHM value of 3.0 cm^–1^ was used to broaden individual peaks. Only modes above 750 cm^–1^ are included. Panel (b) displays the part of the spectrum with the highest IR absorption.

**Figure 4:**
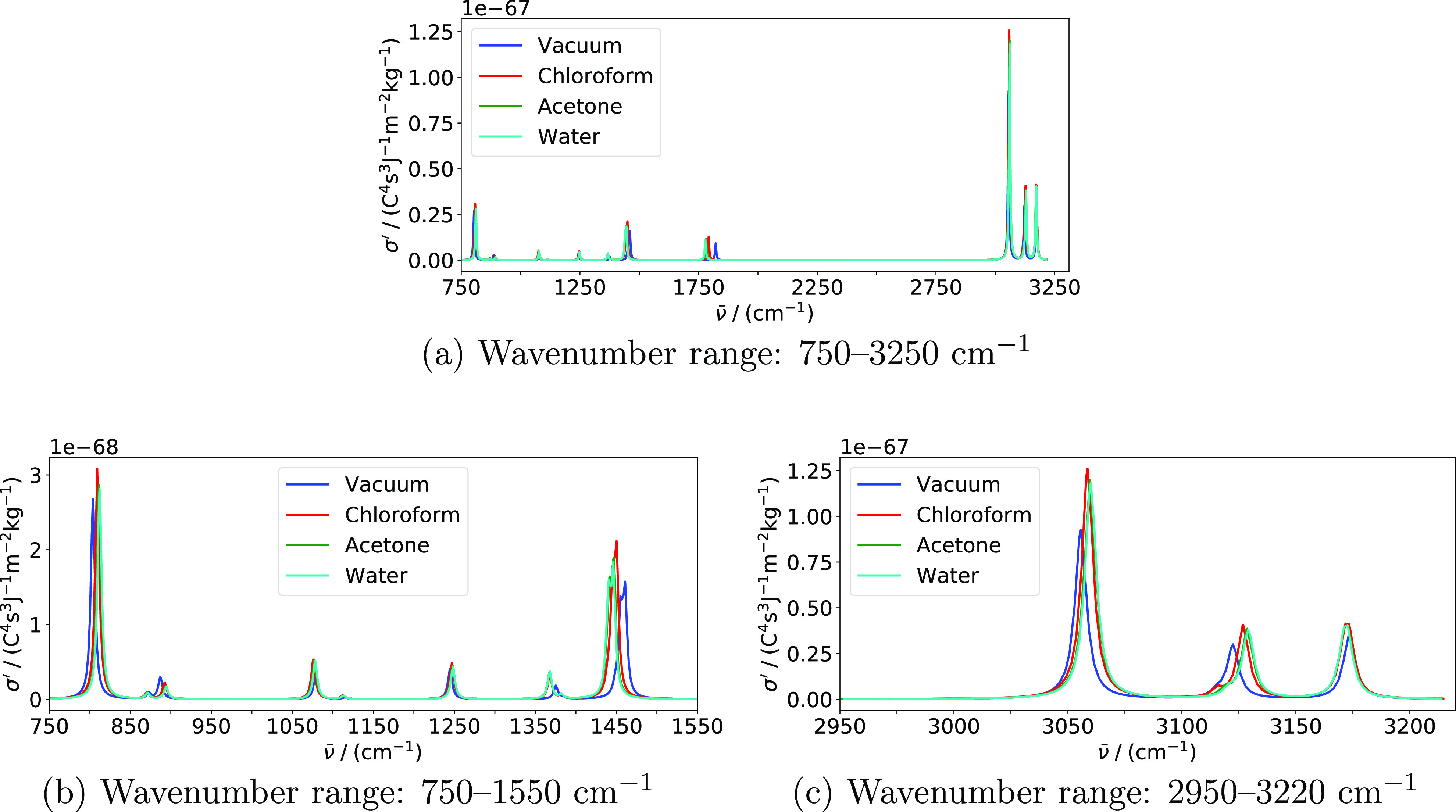
Raman spectra of acetone in various environments modeled using the PCM. Calculations were performed using PCM-PBE0/pcseg-2 using an input wavelength of 514.5 nm. An HWHM value of 3.0 cm^–1^ was used to broaden individual peaks. Only modes above 750 cm^–1^ are included. Panels (b,c) display the parts of the spectrum with the highest Raman activity.

IR and Raman spectra for acetone in vacuum and in the presence of solvents modeled using the PCM are shown in Figures 3 and 4.

When comparing these results with the corresponding spectra obtained using PE to model solvent effects (Figures 1 and 2), there are two substantial qualitative differences. First, the PCM is not able to reproduce the inhomogeneous broadening due to lack of explicit configurational sampling. These effects are substantial in the spectra calculated with PE, especially for water. Second, the effect of the acetone solvent is very similar to the effect of chloroform when modeled using the PE model but similar to the effect of water when modeled using the PCM. The same trend is observed in the bond lengths (Table 3).

Solvent shifts of IR- and Raman-active modes modeled using the PCM are qualitatively similar to those modeled using the PE model, although there are some differences. The carbonyl stretching mode is shifted by −43 cm^–1^ from vacuum to water using the PCM, which is 10 cm^–1^ less than using the PE model. The acetone solvent shift of the carbonyl stretch (−41 cm^–1^) is similar to the water solvent shift using the PCM, whereas it is only −21 cm^–1^ using the PE model. For the C–C stretching mode, the PCM predicts comparatively small shifts for all solvents, whereas the PE model predicts a much larger shift in water. Indeed, the asymmetric and symmetric modes are shifted +3 and +8 cm^–1^ with the PCM and +29 and +23 cm^–1^ with the PE model in water, +3 and +7 cm^–1^ with the PCM and +6 and +3 cm^–1^ with the PE model in acetone, and +2 and +5 cm^–1^ with the PCM and +6 and +3 cm^–1^ with the PE model in chloroform. The opposite behavior is observed for the asymmetric methyl deformation mode. None of the solvents cause a shift of this mode using the PE model, whereas the solvents shifts using the PCM model are −15 cm^–1^ in water and acetone and slightly less in chloroform.

The intensity of a peak is measured as the integral of the area under the peak and directly comparing heights between PE and PCM spectra can therefore be misleading. It is more sensible to compare PCM intensities to PE intensities that are averaged over the snapshots (see the Supporting Information). The most prominent change in intensity upon solvation is exhibited by the IR intensity of the carbonyl stretch in water, with an increase of 88% with the PCM and 51% with PE. In general, changes in intensities upon solvation are more pronounced using the PCM than using the PE model. Indeed, the PCM gives larger intensities than PE for all modes except the symmetric C–H stretch, where the intensity is lowered by 22% with the PCM and by 10% with PE.

## 5 Conclusions

We have presented the theory for the calculation of harmonic IR and Raman spectra of embedded molecules using the PE model to describe environment effects. The derived first- and second-order geometric derivatives of the energy and first-order geometric derivative of the dipole and polarizability are fully analytic and have been implemented in a general open-ended framework, thus facilitating extensions to higher-order geometric derivatives.

The implementation is illustrated through proof-of-principle calculations of IR and Raman spectra for acetone in three different solvents, namely, water, acetone, and chloroform. As expected, we observe that the presence of a solvent has a substantial effect on the IR and Raman spectra. This can be observed as frequency shifts, changes in intensities, and broadening and alterations of the shape of the peaks. The effects of hydrogen bonding between the acetone solute and water as a solvent are evident especially from substantial shift and broadening of the carbonyl stretching mode in the IR spectrum and the C–C symmetric stretching mode in the Raman spectrum. These specific solute–solvent effects on the IR and Raman spectra can only be modeled with an atomistic description of the molecular environment. Apart from these specific interactions, comparison of calculations with the PCM and the PE model shows qualitatively similar solvent effects, but in general larger frequency shifts with the PE model and larger intensity changes with the PCM.

This work is the first step toward modeling accurate vibrational spectra in realistic molecular environments. An extension of the present work to higher-order geometric derivatives is in progress. This will allow us to include second-order anharmonic effects through the calculation of cubic and quartic force fields. Moreover, the combination of the current implementation with higher-order electric derivatives^62^ will enable the calculation of, for example, hyperpolarizability gradients and thus hyper-Raman spectroscopy. We will also explore the incorporation of local field effects through an extension of the effective external field model.^116,117^

## Figures and Tables

**Figure A2:**
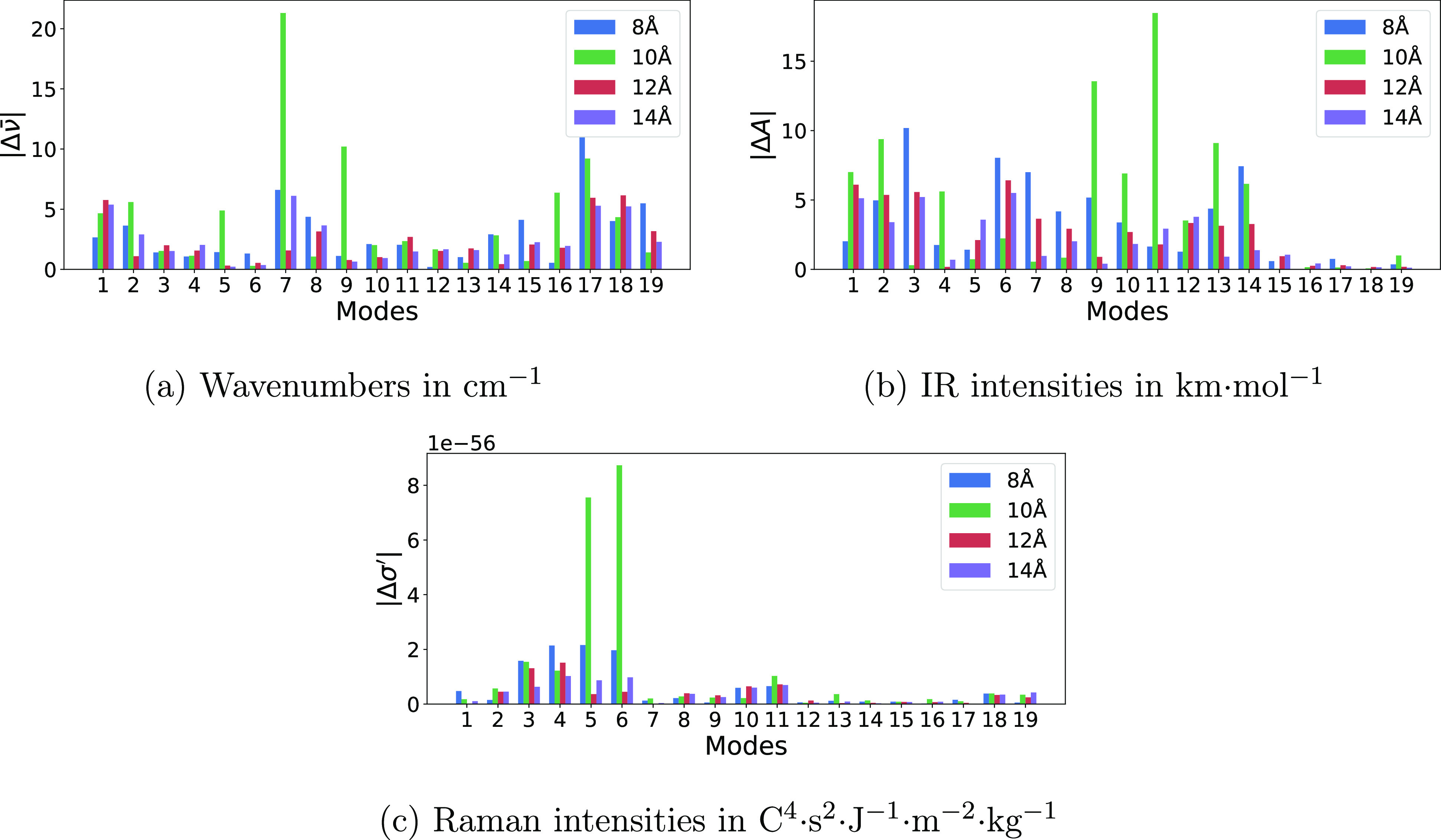
Convergence with respect to size of the molecular environment. Plots show absolute errors of wavenumbers and associated IR and Raman intensities for all vibrational modes above 750 cm^–1^ compared to a 16 Å solvent shell. All calculations were performed using PBE0/pcseg-2 on a single snapshot of acetone embedded in water.

**Figure A3:**
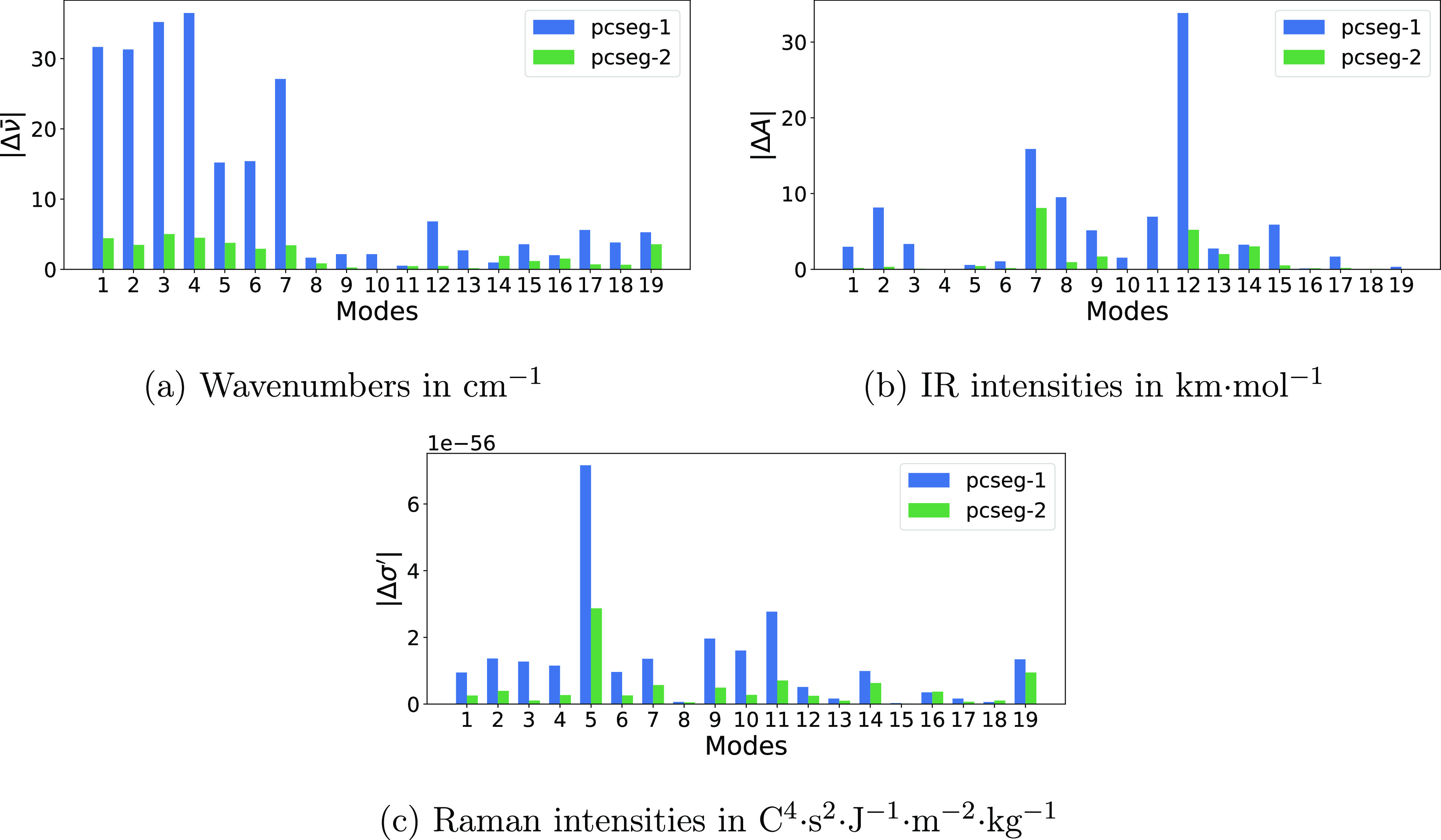
Convergence with respect to the basis set. Plots show absolute errors of wavenumbers and associated IR and Raman intensities for all vibrational modes above 750 cm^–1^ compared to pcseg-3. All calculations were performed using PBE0 on a single snapshot of acetone embedded in a 10 Å water shell.

## Appendix

### Convergence Analyses

We performed a series of convergence analyses with the aim of determining the basis set, size of the environment, and number of snapshots that give an accurate representation of the investigated systems at a reasonable computational cost. For these analyses, we use the acetone-in-water system, since the aqueous environment was found to give the largest solvent effects of the solvents investigated here. To evaluate which basis set and cutoff radius to use, we inspect the absolute error relative to a reference value which is the largest basis set and cutoff radius used. To determine the number of snapshots to include, we investigate the mean and maximum absolute errors (MAE and MAX) of moving averages using samples of different sizes relative to an average value obtained using 250 snapshots. For a sample size *S* and a set of properties *p*_1_, *p*_2_, ..., *p*_*N*_, where *N* is the total number of snapshots, the sample average can be defined as

55

where *j* is the sample index, for example, for *j* = 0, the sample includes properties *p*_1_ to *p*_*S*_, for *j* = 1, it includes properties *p*_2_ to *p*_*S*+1_, and so forth. For a given *S*, *p̅*_*S*_^*j*^ can only be determined for *j* ≤ *N* – *S*. The MAE for a given sample size is then found as

56

where *N*_*S*_ is the number of samples of size *S* and *p̅*_250_ is the global average, that is, the average value across all snapshots. The MAX for a given sample size is determined as the sample average that is the furthest from the global average.

#### A.1 Convergence with Sample Size

In this section, we investigate the convergence with respect to sample size. The purpose is to determine how many snapshots that are needed to reach an error that does not add substantially to the errors introduced by the choice of basis set and system size. For this analysis, we consider the MAE and MAX of moving averages calculated for increasing sample sizes relative to a sample size of 250 snapshots. This gives an indication of the error that can be expected from sampling a number of snapshots consecutively from an MD trajectory. Due to the large number of snapshots, these calculations were performed at the HF/pcseg-1 level of theory in a 12 Å solvent shell of water. The results are presented in Figure A1. We observe a rather slow but steady convergence as the sample size is increased. The carbonyl stretching mode (no. 7 in Figure A1) has the largest error both in terms of wavenumbers and IR intensity but has a very low Raman cross section. Even with 150 snapshots, the MAEs for this mode are 1.0 cm^–1^ and 2.5 km·mol^–1^ for wavenumbers and IR intensity, respectively, and the MAXs are 2.0 cm^–1^ and 5.0 km·mol^–1^, which is of the same order as the basis set error (see Section A.3). The Raman intensities, on the other hand, are reasonably well-converged with a sample size of about 75 snapshots with MAE and MAX below 1.9 × 10^–57^ and 5.0 × 10^–57^ C^4^·s^2^·J^–1^·m^–2^·kg^–1^, respectively, which is well below the largest basis set error (see Section A.3). The convergence of Raman spectra with sample size has been studied previously in the context of Raman optical activity and much more simplistic QM/MM modeling,^118^ where it was concluded that in view of the expected experimental errors in Raman intensities, 50 snapshots were required to give reliable Raman intensities. On this basis and considering the computational cost, we will use a sample size of 50 to calculate IR and Raman spectra, keeping in mind that this may result in comparatively large errors for wavenumbers and IR intensities for some of the modes and in particular for the carbonyl stretching mode.

#### A.2 Convergence with Size of a Molecular Environment

To determine a suitable size of the solvent environment, calculations were performed for a single snapshot of aqueous acetone with a solvent shell radius ranging from 8 to 16 Å. The PBE0 functional was used together with the pcseg-2 basis set in all calculations. As can be seen from the absolute errors presented in Figure A2, none of the properties converge smoothly with system size. In fact, there appears to be an oscillating behavior for some of the modes. From a visual inspection, it is found that the main reason for this oscillating behavior is the change in the position of the acetone molecule within the solvent cavity, whereas the geometry of acetone remains fairly similar for all environments. In our current implementation, the computational cost grows rather steeply with increasing system size, mandating the need to balance cost to errors due to truncation of the size of the environment. Given this limitation, we find that a system size of 12 Å has residual errors that are of the same order as the errors due to our use of the pcseg-2 basis set (see Section A.3). Indeed, root-mean-squared deviations (RMSDs) relative to 16 Å decrease from 6.5 cm^–1^, 6.8 km·mol^–1^, and 2.7 × 10^–56^ C^4^·s^2^·J^–1^·m^–2^·kg^–1^ for 10 Å to 2.9 cm^–1^, 3.3 km·mol^–1^, and 5.6 × 10^–57^ C^4^·s^2^·J^–1^·m^–2^·kg^–1^ for 12 Å. Increasing the system size to 14 Å does not improve the overall error (RMSDs relative to 16 Å are 3.0 cm^–1^, 2.8 km·mol^–1^, and 5.0 × 10^–57^ C^4^·s^2^·J^–1^·m^–2^·kg^–1^).

#### A.3 Convergence with the Basis Set

In order to identify the most accurate and cost-efficient basis set to be used in the calculation of the vibrational properties, vibrational frequencies and associated IR and Raman intensities were calculated for a single snapshot of aqueous acetone using PBE0 with three different basis sets: pcseg-1, pcseg-2, and pcseg-3^107^ that are of double-, triple-, and quadruple-ζ quality, respectively. The frequency-dependent polarizability derivatives were evaluated at a wavelength of 514.5 nm. The size of the environment was arbitrarily set to include solvent molecules with a center of mass within a 10 Å radius from the center of mass of the acetone molecule. The results can be seen in Figure A3 as absolute errors compared to results obtained using pcseg-3. Wavenumbers (Figure A3a) are off by up to 37 cm^–1^ for the pcseg-1 basis set. In contrast, the largest errors obtained using pcseg-2 are about 3–5 cm^–1^ and are mostly associated with the high-frequency modes, that is, C–H and C=O stretching modes.^115^ In terms of intensities, using pcseg-1 results in errors that are generally below 10 km·mol^–1^ for IR (Figure A3b) and below 3 × 10^–56^ C^4^·s^2^·J^–1^·m^–2^·kg^–1^ for Raman (Figure A3c). Using pcseg-2 results in errors that are generally below 4 km·mol^–1^ for IR and below 0.7 × 10^–56^ C^4^·s^2^·J^–1^·m^–2^·kg^–1^ for Raman. A few vibrational modes dominate, showing larger errors for both basis sets. Specifically, the IR intensities of the C=O stretch and symmetric CH_3_ deformation modes are off by about 8.1 and 5.2 km·mol^–1^, respectively, whereas in the Raman case, the symmetric C–H and C–C stretching modes are off by 2.9 × 10^–56^ and 0.9 × 10^–56^ C^4^·s^2^·J^–1^·m^–2^·kg^–1^, respectively, for the pcseg-2 basis set. These are the modes with the largest intensities and absorption cross sections. Overall, using pcseg-2 results in RMSDs of 2.7 cm^–1^, 2.4 km·mol^–1^, and 0.77 × 10^–56^ C^4^·s^2^·J^–1^·m^–2^·kg^–1^, whereas pcseg-1 results in RMSDs of 17.6 cm^–1^, 9.5 km·mol^–1^, and 2.0 × 10^–56^ C^4^·s^2^·J^–1^·m^–2^·kg^–1^, both compared to pcseg-3. Our results clearly show that pcseg-1 is not adequate for accurate calculations of IR and Raman intensities. Taking into account that the relative computational cost of pcseg-2 compared to pcseg-3 is approximately one-fourth, we conclude that pcseg-2 is a good compromise between accuracy and cost.

## Supporting Information Available

Tabulated wavenumbers and associated IR and Raman intensities for acetone in vacuum and in chloroform, acetone, and water solutions and absolute differences between wavenumbers calculated with and without projecting out rotation and translation (PDF)
