# Supplementary material for: Harmonic Infrared and Raman Spectra in Molecular Environments Using the Polarizable Embedding Model
Source: J Chem Theory Comput. 2021 May 19;17(6):3599–617. doi: 10.1021/acs.jctc.0c01323 (PMC8278393; doi:10.1021/acs.jctc.0c01323)
Supplement: Supplementary file 1 — ct0c01323_si_001.pdf [file ct0c01323_si_001.pdf]

Supporting Information: Harmonic Infrared and Raman Spectra in Molecular Environments using the Polarizable Embedding Model

Karen Oda Hjorth Minde Dundas,[†] Maarten T. P. Beerepoot,[†] Magnus Ringholm,[†] Simen Reine,[‡] Radovan Bast,[¶] Nanna Holmgaard List,[§,‖] Jacob Kongsted,[⊥] Kenneth Ruud,[*,†] and Jógvan Magnus Haugaard Olsen[*,†,#]

†*Hylleraas Centre for Quantum Molecular Sciences, Department of Chemistry, UiT The Arctic University of Norway, N-9037 Tromsø, Norway*

‡*Hylleraas Centre for Quantum Molecular Sciences, Department of Chemistry, University of Oslo, N-0315 Oslo, Norway*

¶*Department of Information Technology, UiT The Arctic University of Norway, N-9037 Tromsø, Norway*

§*Department of Chemistry and The PULSE Institute, Stanford University, Stanford, CA 94305, USA*

‖*SLAC National Accelerator Laboratory, Menlo Park, CA 94025, USA*

⊥*Department of Physics, Chemistry and Pharmacy, University of Southern Denmark, DK–5230 Odense M, Denmark*

#*Present address: Department of Chemistry, Technical University of Denmark, DK-2800 Kgs. Lyngby, Denmark*

E-mail: kenneth.ruud@uit.no; jmho@kemi.dtu.dk

Table S1: Averaged Wavenumbers and Associated IR and Raman Intensities of Acetone in Water Modelled by the PE Model[a]

Mode	Wavenumber[b]	IR[c]	Raman ($\lambda_0 = 0$ nm)[d]	Raman ($\lambda_0 = 514.5$ nm)[e]
1	3193 (16.7)	4.561 (4.4)	44.281 (5.3)	33.956 (3.6)
2	3176 (13.0)	5.534 (4.3)	38.883 (5.8)	30.618 (4.4)
3	3136 (8.4)	3.124 (2.5)	46.876 (7.5)	39.472 (6.4)
4	3124 (7.6)	2.363 (2.2)	33.462 (8.9)	28.716 (7.5)
5	3063 (7.9)	4.293 (3.7)	159.816 (29.6)	148.469 (28.0)
6	3051 (6.6)	3.958 (3.1)	41.747 (29.7)	39.882 (28.1)
7	1769 (19.8)	303.181 (22.7)	1.307 (0.2)	18.107 (1.7)
8	1486 (6.9)	24.907 (5.5)	0.138 (0.1)	3.236 (2.3)
9	1470 (4.9)	23.022 (14.1)	0.677 (0.2)	17.014 (4.8)
10	1458 (4.6)	12.528 (11.7)	0.440 (0.2)	11.444 (5.0)
11	1448 (5.1)	22.367 (14.8)	0.598 (0.2)	16.111 (5.4)
12	1399 (7.7)	99.709 (8.6)	0.136 (0.1)	4.582 (2.7)
13	1383 (5.7)	34.325 (9.1)	0.169 (0.1)	6.188 (2.1)
14	1276 (9.9)	52.941 (9.5)	0.114 (0.0)	6.129 (1.0)
15	1119 (5.6)	10.961 (2.8)	0.015 (0.0)	1.369 (0.6)
16	1088 (6.2)	2.285 (2.2)	0.103 (0.0)	9.578 (1.5)
17	931 (16.2)	4.037 (1.2)	0.015 (0.0)	2.182 (1.0)
18	881 (9.4)	1.034 (0.7)	0.023 (0.0)	4.847 (2.8)
19	828 (8.6)	0.983 (0.5)	0.121 (0.0)	34.538 (5.0)

[a] standard deviation in parentheses; [b] given in units of cm^{-1}; [c] given in units of $km \cdot mol^{-1}$; [d] given in units of $C^4 \cdot s^2 \cdot J^{-1} \cdot m^{-2} \cdot kg^{-1}$ [$\times 10^{-60}$]; [e] given in units of $C^4 \cdot s^2 \cdot J^{-1} \cdot m^{-2} \cdot kg^{-1}$ [$\times 10^{-57}$].

Table S2: Averaged Wavenumbers and Associated IR and Raman Intensities of Acetone in Acetone Modelled by the PE Model[a]

Mode	Wavenumber[b]	IR[c]	Raman ($\lambda_0 = 0$ nm)[d]	Raman ($\lambda_0 = 514.5$ nm)[e]
1	3181 (6.2)	5.338 (1.5)	48.406 (2.6)	37.958 (2.0)
2	3173 (4.1)	8.960 (2.6)	43.443 (2.6)	34.700 (2.1)
3	3130 (6.1)	5.638 (1.7)	56.105 (7.8)	47.970 (6.9)
4	3121 (3.7)	1.685 (1.0)	24.196 (9.5)	21.001 (8.1)
5	3060 (4.7)	1.623 (1.1)	190.095 (20.8)	176.771 (19.7)
6	3053 (2.9)	0.479 (0.5)	19.731 (20.2)	18.842 (19.0)
7	1806 (6.0)	247.751 (8.1)	1.567 (0.1)	19.581 (0.8)
8	1481 (3.5)	24.251 (1.8)	0.059 (0.0)	1.401 (1.2)
9	1465 (3.4)	22.735 (10.2)	0.878 (0.1)	22.294 (3.1)
10	1455 (3.0)	9.205 (8.5)	0.591 (0.2)	15.329 (5.7)
11	1451 (1.8)	8.889 (7.5)	0.446 (0.2)	11.818 (6.4)
12	1389 (2.7)	91.660 (2.9)	0.043 (0.0)	1.346 (0.3)
13	1378 (2.6)	26.580 (3.2)	0.099 (0.0)	3.715 (0.6)
14	1254 (2.9)	63.157 (3.6)	0.134 (0.0)	8.005 (0.6)
15	1116 (2.5)	5.982 (0.8)	0.009 (0.0)	0.881 (0.2)
16	1083 (3.5)	0.132 (0.1)	0.094 (0.0)	8.827 (0.7)
17	906 (6.7)	5.707 (0.8)	0.024 (0.0)	4.211 (0.9)
18	876 (4.4)	0.543 (0.4)	0.015 (0.0)	3.193 (1.2)
19	812 (4.1)	1.547 (0.3)	0.151 (0.0)	47.481 (3.1)

[a] standard deviation in parentheses; [b] given in units of cm^{-1}; [c] given in units of $km{\cdot}mol^{-1}$; [d] given in units of $C^4{\cdot}s^2{\cdot}J^{-1}{\cdot}m^{-2}{\cdot}kg^{-1}$ [$\times 10^{-60}$]; [e] given in units of $C^4{\cdot}s^2{\cdot}J^{-1}{\cdot}m^{-2}{\cdot}kg^{-1}$ [$\times 10^{-57}$].

Table S3: Averaged Wavenumbers and Associated IR and Raman Intensities of Acetone in Chloroform Modelled by the PE Model[a]

Mode	Wavenumber[b]	IR[c]	Raman ($\lambda_0 = 0$ nm)[d]	Raman ($\lambda_0 = 514.5$ nm)[e]
1	3181 (6.3)	5.420 (1.7)	48.503 (2.8)	38.025 (2.2)
2	3173 (4.2)	8.721 (2.5)	43.437 (2.5)	34.684 (2.0)
3	3123 (6.3)	5.608 (1.6)	55.379 (7.0)	47.352 (6.2)
4	3121 (3.8)	1.819 (1.1)	25.008 (8.8)	21.702 (7.5)
5	3060 (4.8)	1.654 (1.1)	190.269 (21.0)	176.977 (19.9)
6	3053 (2.9)	0.462 (0.4)	19.545 (20.7)	18.658 (19.4)
7	1806 (6.1)	247.500 (8.0)	1.568 (0.1)	19.591 (0.8)
8	1481 (3.3)	24.193 (1.9)	0.061 (0.0)	1.431 (1.2)
9	1466 (3.3)	21.915 (9.7)	0.868 (0.1)	22.023 (3.1)
10	1456 (3.1)	9.627 (8.0)	0.593 (0.2)	15.365 (5.4)
11	1451 (1.8)	9.145 (7.4)	0.451 (0.2)	11.959 (6.2)
12	1389 (2.8)	91.587 (3.0)	0.043 (0.0)	1.328 (0.4)
13	1378 (2.7)	26.531 (3.0)	0.099 (0.0)	3.712 (0.6)
14	1254 (3.0)	63.353 (3.7)	0.134 (0.0)	7.977 (0.5)
15	1116 (2.4)	6.007 (0.7)	0.009 (0.0)	0.896 (0.2)
16	1082 (3.1)	0.131 (0.1)	0.094 (0.0)	8.819 (0.7)
17	906 (6.8)	5.812 (0.8)	0.024 (0.0)	4.245 (0.9)
18	876 (4.5)	0.524 (0.4)	0.016 (0.0)	3.294 (1.2)
19	812 (3.8)	1.555 (0.3)	0.151 (0.0)	47.513 (3.1)

[a] standard deviation in parentheses; [b] given in units of cm^{-1}; [c] given in units of $km{\cdot}mol^{-1}$; [d] given in units of $C^4{\cdot}s^2{\cdot}J^{-1}{\cdot}m^{-2}{\cdot}kg^{-1}$ [$\times 10^{-60}$]; [e] given in units of $C^4{\cdot}s^2{\cdot}J^{-1}{\cdot}m^{-2}{\cdot}kg^{-1}$ [$\times 10^{-57}$].

Table S4: Wavenumbers and Associated IR and Raman Intensities of Acetone in Vacuum

Mode	Wavenumber[a]	IR[b]	Raman ($\lambda_0 = 0$ nm)[c]	Raman ($\lambda_0 = 514.5$ nm)[d]
1	3175	4.496	45.151	35.311
2	3173	9.558	35.165	28.219
3	3123	11.851	60.138	51.772
4	3116	0.000	6.743	6.144
5	3056	4.817	177.032	164.416
6	3050	0.556	0.646	0.813
7	1824	200.895	1.390	16.493
8	1481	23.805	0.001	0.006
9	1462	31.168	0.922	23.635
10	1455	0.000	0.737	18.653
11	1449	0.737	0.076	2.149
12	1388	80.149	0.019	0.501
13	1377	21.572	0.073	2.842
14	1248	63.346	0.117	7.148
15	1116	3.473	0.004	0.427
16	1080	0.041	0.069	6.421
17	888	5.988	0.026	5.167
18	874	0.000	0.008	1.400
19	808	1.630	0.147	47.177

[a] given in units of cm^{-1}; [b] given in units of $km \cdot mol^{-1}$; [c] given in units of $C^4 \cdot s^2 \cdot J^{-1} \cdot m^{-2} \cdot kg^{-1}$ [$\times 10^{-60}$]; [d] given in units of $C^4 \cdot s^2 \cdot J^{-1} \cdot m^{-2} \cdot kg^{-1}$ [$\times 10^{-57}$].

Table S5: Wavenumbers and Associated IR and Raman Intensities of Acetone in Water Modelled by the PCM

Mode	Wavenumber[a]	IR[b]	Raman ($\lambda_0 = 0$ nm)[c]	Raman ($\lambda_0 = 514.5$ nm)[d]
1	3173	8.333	81.628	43.498
2	3171	20.217	69.652	35.484
3	3129	8.581	129.258	65.803
4	3121	0.000	10.321	7.343
5	3060	1.185	376.677	209.725
6	3052	0.653	0.490	0.846
7	1779	375.709	2.584	20.111
8	1463	33.958	0.000	0.006
9	1446	59.376	1.685	27.546
10	1440	0.000	1.157	20.731
11	1438	3.565	0.179	2.873
12	1382	121.601	0.048	1.007
13	1368	50.516	0.294	6.463
14	1248	81.062	0.192	7.728
15	1113	9.859	0.024	0.776
16	1078	0.058	0.159	9.117
17	894	7.404	0.019	2.819
18	871	0.000	0.011	1.339
19	812	2.403	0.212	49.909

[a] given in units of cm^{-1}; [b] given in units of $km{\cdot}mol^{-1}$; [c] given in units of $C^4{\cdot}s^2{\cdot}J^{-1}{\cdot}m^{-2}{\cdot}kg^{-1}$ [$\times10^{-60}$]; [d] given in units of $C^4{\cdot}s^2{\cdot}J^{-1}{\cdot}m^{-2}{\cdot}kg^{-1}$ [$\times10^{-57}$].

Table S6: Wavenumbers and Associated IR and Raman Intensities of Acetone in Acetone Modelled by the PCM

Mode	Wavenumber[a]	IR[b]	Raman ($\lambda_0 = 0$ nm)[c]	Raman ($\lambda_0 = 514.5$ nm)[d]
1	3173	7.912	79.175	44.125
2	3171	19.369	66.795	35.899
3	3129	8.750	123.709	66.988
4	3119	0.000	10.104	7.400
5	3060	1.391	359.405	212.864
6	3051	0.586	0.580	0.910
7	1781	362.994	2.512	20.651
8	1463	33.059	0.000	0.007
9	1447	57.434	1.655	28.468
10	1441	0.000	1.132	21.157
11	1438	3.497	0.169	2.903
12	1382	118.933	0.046	0.992
13	1367	47.286	0.264	6.269
14	1248	80.024	0.187	7.920
15	1113	9.163	0.023	0.797
16	1077	0.058	0.147	9.169
17	894	7.280	0.020	3.015
18	871	0.000	0.011	1.383
19	811	2.297	0.209	50.913

[a] given in units of cm^{-1}; [b] given in units of $km \cdot mol^{-1}$; [c] given in units of $C^4 \cdot s^2 \cdot J^{-1} \cdot m^{-2} \cdot kg^{-1}$ [$\times 10^{-60}$]; [d] given in units of $C^4 \cdot s^2 \cdot J^{-1} \cdot m^{-2} \cdot kg^{-1}$ [$\times 10^{-57}$].

Table S7: Wavenumbers and Associated IR and Raman Intensities of Acetone in Chloroform Modelled by the PCM

Mode	Wavenumber[a]	IR[b]	Raman ($\lambda_0 = 0$ nm)[c]	Raman ($\lambda_0 = 514.5$ nm)[d]
1	3173	6.571	69.779	46.116
2	3171	16.264	56.795	37.421
3	3127	9.443	103.910	71.013
4	3116	0.000	9.300	7.604
5	3059	2.252	299.749	223.322
6	3049	0.460	0.795	1.056
7	1792	315.271	2.212	22.504
8	1465	30.509	0.000	0.007
9	1450	48.920	1.479	31.352
10	1445	0.000	1.035	22.684
11	1439	2.602	0.137	3.109
12	1382	109.084	0.035	0.881
13	1368	38.450	0.191	5.851
14	1247	75.909	0.168	8.579
15	1112	6.796	0.015	0.857
16	1076	0.040	0.118	9.367
17	892	6.859	0.024	3.868
18	871	0.000	0.010	1.558
19	809	1.919	0.196	54.698

[a] given in units of cm^{-1}; [b] given in units of $km{\cdot}mol^{-1}$; [c] given in units of $C^4{\cdot}s^2{\cdot}J^{-1}{\cdot}m^{-2}{\cdot}kg^{-1}$ [$\times10^{-60}$]; [d] given in units of $C^4{\cdot}s^2{\cdot}J^{-1}{\cdot}m^{-2}{\cdot}kg^{-1}$ [$\times10^{-57}$].

Table S8: Wavenumbers and Associated IR and Raman Intensities of Acetone in Vacuum Calculated with Gaussian

Mode	Wavenumber[a]	IR[b]	Raman ($\lambda_0 = 0$ nm)[c]	Raman ($\lambda_0 = 514.5$ nm)[d]
1	3174	4.524	45.283	35.363
2	3173	9.559	35.149	28.147
3	3122	11.881	60.095	51.674
4	3116	0.000	6.742	6.130
5	3056	4.886	176.814	163.890
6	3049	0.562	0.649	0.814
7	1822	199.965	1.376	16.380
8	1479	23.771	0.001	0.005
9	1461	30.895	0.916	23.519
10	1455	0.000	0.737	18.627
11	1449	0.640	0.077	2.174
12	1384	82.688	0.015	0.398
13	1375	22.729	0.081	3.147
14	1245	60.844	0.117	7.169
15	1115	3.476	0.004	0.428
16	1079	0.036	0.069	6.416
17	887	5.888	0.026	5.168
18	873	0.000	0.008	1.385
19	804	1.520	0.145	47.607

[a] given in units of cm^{-1}; [b] given in units of $km \cdot mol^{-1}$; [c] given in units of $C^4 \cdot s^2 \cdot J^{-1} \cdot m^{-2} \cdot kg^{-1}$ [$\times 10^{-60}$]; [d] given in units of $C^4 \cdot s^2 \cdot J^{-1} \cdot m^{-2} \cdot kg^{-1}$ [$\times 10^{-57}$].

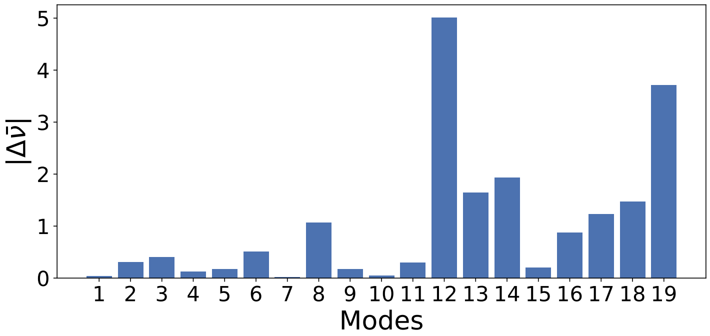

(a) Chloroform

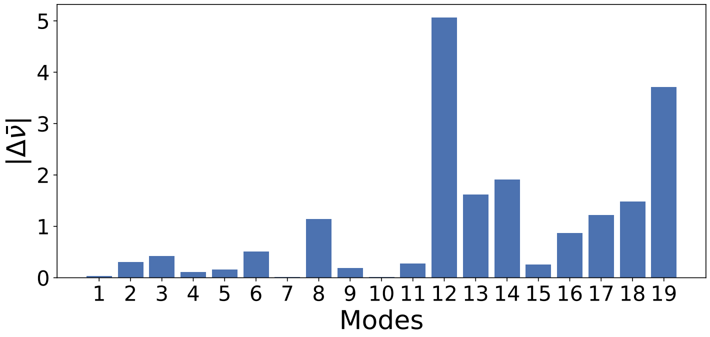

(b) Acetonitrile

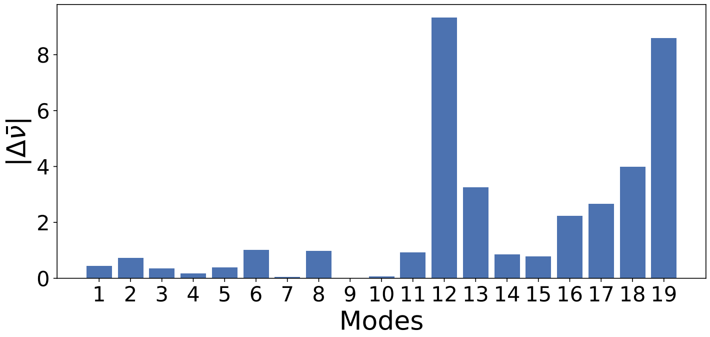

(c) Water

Figure S1: Absolute differences (in cm^{-1}) between average wavenumbers calculated with and without projecting out translation and rotation.
